# Tongue Images Classification Based on Constrained High Dispersal Network

**DOI:** 10.1155/2017/7452427

**Published:** 2017-03-30

**Authors:** Dan Meng, Guitao Cao, Ye Duan, Minghua Zhu, Liping Tu, Dong Xu, Jiatuo Xu

**Affiliations:** ^1^MOE Research Center for Software/Hardware Co-Design Engineering, East China Normal University, Shanghai 200062, China; ^2^Department of Computer Science, University of Missouri, Columbia, MO 65211, USA; ^3^Department of TCM Information and Technology Center, Shanghai University of TCM, Shanghai, China; ^4^Department of Basic Medical College, Shanghai University of Traditional Chinese Medicine, 1200 Cailun Road, Pudong New Area, Shanghai 201203, China

## Abstract

Computer aided tongue diagnosis has a great potential to play important roles in traditional Chinese medicine (TCM). However, the majority of the existing tongue image analyses and classification methods are based on the low-level features, which may not provide a holistic view of the tongue. Inspired by deep convolutional neural network (CNN), we propose a novel feature extraction framework called constrained high dispersal neural networks (CHDNet) to extract unbiased features and reduce human labor for tongue diagnosis in TCM. Previous CNN models have mostly focused on learning convolutional filters and adapting weights between them, but these models have two major issues: redundancy and insufficient capability in handling unbalanced sample distribution. We introduce high dispersal and local response normalization operation to address the issue of redundancy. We also add multiscale feature analysis to avoid the problem of sensitivity to deformation. Our proposed CHDNet learns high-level features and provides more classification information during training time, which may result in higher accuracy when predicting testing samples. We tested the proposed method on a set of 267 gastritis patients and a control group of 48 healthy volunteers. Test results show that CHDNet is a promising method in tongue image classification for the TCM study.

## 1. Introduction

Tongue image classification is a key component in traditional Chinese medicine (TCM). For thousands of years, Chinese medical physicians have judged the patient's health status by examining the tongue's color, shape, and texture [1, 2]. With the improvement in digital medical imaging equipment and pattern recognition methods, computer aided tongue diagnoses have a great potential to play an important role in TCM by providing more accurate, consistent, and objective clinical diagnoses [3].

In the past decades, tongue image feature extraction methods have been intensively studied. According to these studies, computer aided tongue diagnosis methods can be divided into two categories: single feature and multifeatures. Many single feature extraction methods have been proposed and applied to tongue images analysis. Such methods can exploit useful information based on a simple descriptor such as color, texture, shape, and orientation. As presented in [4–9], a single feature was used to analyze the tongue images. Li and Yuen [4] investigated and reported the color matching of tongue images with different metrics in different color space. In [5], Wang et al. presented a color recognition scheme of tongue images by obtaining a number of homogenous regions before classification. Spectral Angle Mapper (SAM) [6] can recognize and classify tongue colors using their spectral signatures rather than their color values in RGB color space. In [7] the authors aimed to build a mathematically described tongue color space for diagnostic feature extraction based on the statistical distribution of tongue color. In [8], partition patients' state (either healthy or diseased) was quantitatively analyzed using geometry tongue shape features with the computerized methods. In [9], Cao et al. presented a feature extraction method based on statistical features.

Although many models based on the strategy of a single feature have been proposed and achieved successful results, this type of method only utilizes low-level features. So multifeatures [10, 11] are helpful to detect normal and abnormal tongue images. The works [12–15] used multifeatures (such as the combination of color and texture or shape) to identify and match tongue images. In [12], Kanawong et al. proposed a coating separation step before extracting features. In [13], Guo proposed a color-textured operator called primary difference signal local binary pattern (PDSLBP) to handle the tongue image matching problem. In [14], a tongue computing model (TCoM) based on quantitative measurements that include chromatic and textural features was proposed to diagnose appendicitis. In [15], multilabeled learning was applied to tongue image classification after extracting color and texture features.

In fact, the aforementioned methods only used low-level features either single feature or multifeatures, which cannot completely describe the characteristics of the tongue. It was necessary for us to integrate a framework that can generate complete features from tongue images. Thus, high-level features were necessary for computer aided tongue analysis. Most existing publications have described and applied deep learning models to extract high-level feature representations for a wide range of vision analysis tasks [16] (such as hand-written digit recognition [17], face recognition [18], and object recognition [19]). However, there exists little or no literature on computer aided tongue image analysis using deep learning models, whereas computer aided expert system with unambiguity and objectivity tongue analysis results can be used to facilitate both traditional Chinese and Western medical practices' diagnostic results.

PCANet [23] is the least simple deep neural network in the visual classification task developed by Chan et al. [23]. In [23], PCANet was compared with the well-known convolutional neural networks (CNN) [24] with respect to performance on various tasks. Instead of initializing the weights of the network in CNN randomly or via pretraining and then updating them in a backpropagation way, PCANet [23] treated only the most basic PCA filters for use in the convolution filter bank in each stage without further training. Since PCANet [23] was developed, high-level extraction methods based on PCANet [23] have been studied in the field of face recognition [25], human fall detection [26], speech emotion detection [27], and so forth. Although PCANet has not been applied to the field of computer aided tongue diagnoses, it has several characteristics that make it applicable for tongue image classification. As presented in PCANet [23], it is easy to train and to adapt to different data and tasks without changing the architecture of network. Besides, there is little need to fine-tune parameters. Additionally, PCANet [23] combined with machine-learning classification algorithms, such as *k*-nearest neighbor (KNN), SVM, and Random Forest (RF), can achieve excellent performance in classification tasks.

However, we observed that the original PCANet [23] has two major issues: redundancy and insufficient capability to handle unbalanced samples. The PCA method, by its nature, will respond to the large eigenvalues. Therefore, in the PCANet, there is often a significant amount of redundancy in the convoluted feature maps. Another issue is that classification tasks mentioned in PCANet [23] are based on the assumption that the distribution of samples is balanced and the number of samples in dataset is large. Since PCANet [23] only consists of a convolutional layer and histogram, we had no way of knowing if it could gain sufficient information in distinguishing normal from abnormal status for our specific tongue classification task.

Inspired by the works of deep learning models and their variants, this paper proposes a framework referred to as constrained high dispersal neural networks (CHDNet) based on PCA convolutional kernels to aid in tongue diagnosis. The proposed CHDNet learns useful features from the clinical data in an unsupervised way, and, with these obtained features, a supervised machine-learning technique is used to learn how to partition a patient's health status into normal or abnormal states.

The main contributions of this paper are as follows:

- A new feature extraction method (CHDNet) is presented which explores the feature representations of normal and abnormal tongue images, which mainly benefit from using four important components: nonlinear transformation, multiscale feature analysis, high dispersal, and local normalization.
- CHDNet can provide robust feature representations to predict patient's health status based on obtained samples with unbalanced distribution, given the fact that most people come to the hospital for their physician's advice or prescription when they feel sick.
- CHDNet has been evaluated by using diagnosed samples by clinicians. Experimental results confirmed that gastritis patients classified by our proposed model were in good agreement with the clinician's diagnosis.

The rest of this paper is organized as follows.  Section 2 introduces the framework of the proposed CHDNet. Experimental results are shown in Section 3. Finally, the conclusion and future work are presented in Section 4.

## 2. Algorithm Overview

**Figure 1:**
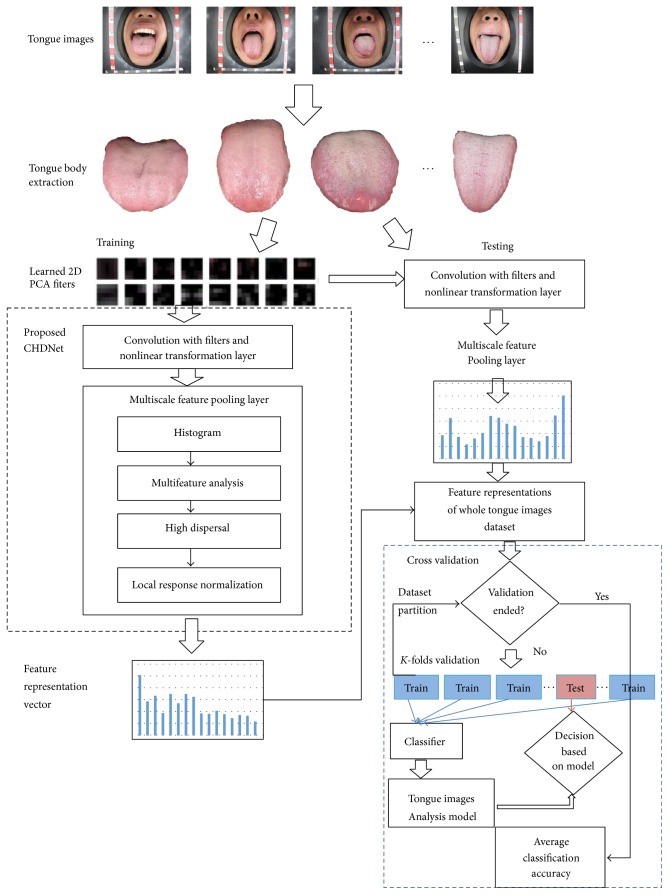
Flowchart of normal and abnormal detection framework based on CHDNet.

For each image, we first extracted the tongue body from its background. Then, we applied CHDNet to learn features of normal and abnormal tongue bodies.  Figure 1 shows the flowchart of normal and abnormal detection framework based on CHDNet. For each tongue image, after extracting the tongue body from its background, it was normalized to fixed height and weight. After these two steps of preprocessing, we partitioned the tongue images into training and testing sets to learn convolutional kernels and generate feature representations. We then sent feature representations of the whole tongue images dataset into classifier using *k* folds cross validation strategy for the classification task. The samples were labeled into two classes, namely, normal and abnormal. The whole feature representations were separated into *k* training and testing sets in *k* different ways. The classifier was first trained with *k* − 1 subsets, and then its performance was evaluated on the *k*th subset. Each subset was used as the test set once through repeating the process *k* times. The final result was obtained by averaging the outcome produced in *k* corresponding rounds.

Algorithm 1 elaborates the details of the proposed CHDNet.

### 2.1. Features Extraction with the Proposed CHDNet

The original tongue image sizes are 1728 × 1296 pixels. After tongue body extraction, we notice that our interest area (tongue body) is around 500 × 500 pixels, so we zoomed all the tongue body images into 32 × 32 pixels.

For image classification tasks, the hand-crafted low-level features can generally work well when dealing with some specific tasks or data processes, like HOG [20], LBP [28], and SIFT [19] for object recognition. Yet they are not universal for all conditions, especially when the application scenario is medicine. Therefore, the concept of learning features from data of interest is proposed to overcome the limitation of hand-crafted features, and deep learning is treated as a better method to extract high-level features, which provide more invariance into intraclass variability. As mentioned in Section 2, PCANet [23] is a deep network, which has the capability to extract high-level features.

Compared to PCANet [23], CHDNet has four important components:

- *High Dispersal.* With the high dispersal operation, features in each feature map achieve the property of dispersal without redundancy.
- *Local Response Normalization*. After the processing of high dispersal, features in the same position of different feature maps still have redundancy. The proposed local response normalization aims to solve this problem.
- *Nonlinear Transformation Layer*. Since we use tanh⁡(*x*) in feature convolutional layer, negative value exists, which conflicts with the principle of visual systems. In order to prepare suitable inputs for convolutional layer and local response normalization, we add a nonlinear transformation layer after each convolution layer.
- *Multiscale Feature Analysis*. To improve the ability to handle deformation, we introduce multiscale feature analysis before high dispersal and local response normalization.

It should be pointed out that these new ideas and methods are uniquely designed to address the major limitations of the original PCANet [23] approach and aim to significantly improve its performance. Experimental results in Section 3 will validate how these new ideas impact the performance of tongue images classification. Since we add several constraints on the networks and features distributed in a high dispersal manner, our proposed feature extraction method is called the constrained high dispersal neural network (CHDNet).

Compared with convolutional neural network (CNN), which is a complex neural network architecture, requiring tricky parameter tuning and time-consuming calculations, CHDNet is extremely simple and efficient.

**Figure 2:**
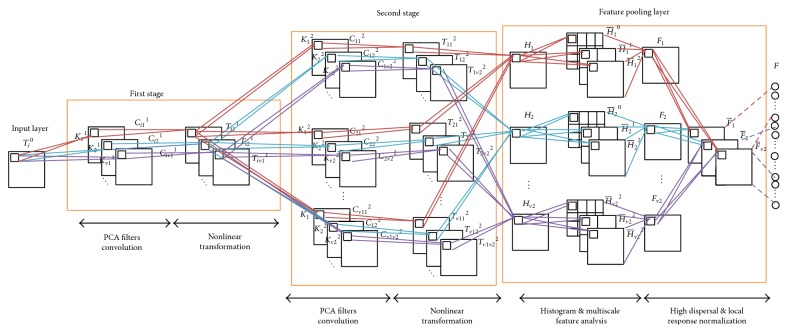
The structure of the two-stage CHDNet.

Figure 2 shows the basic architecture of CHDNet. It is composed of three components: PCA filters convolution layer, nonlinear transformation layer, and a feature pooling layer.

#### 2.1.1. Nonlinear Transformation

**Figure 3:**
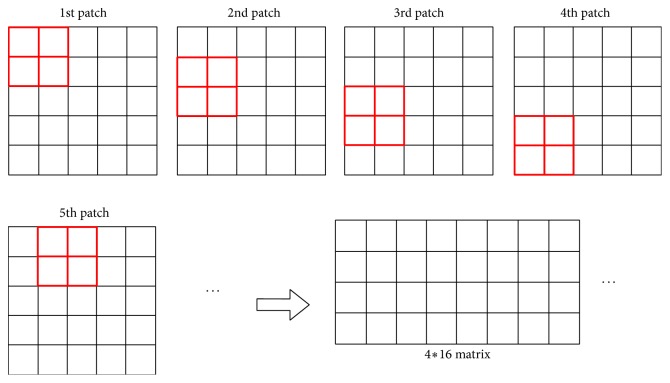
Illustration of 2 × 2 patch size taking for a 5 × 5 image.

Suppose we have *N* training samples of size *m* × *n*. As illustrated in Figure 3, we collect patches of size *s*_1_ × *s*_2_, with stride = 1. We also do patch mean removal on all overlapping patches and, then, vectorize and combine them into a matrix.

For *i*th image *T*_*i*_^0^ at input stage, after patch mean removal operation we get $T_{i}^{0}=[{\bar{t}}_{i,1}^{0},{\bar{t}}_{i,2}^{0},\ldots ,{\bar{t}}_{i,p}^{0}]\in R^{s_{1}s_{2}\times p}$. Similar to *i*th image, for the entire training samples we have {*T*_*i*_^0^}_*i*=1_^*N*^ = [*T*_1_^0^, *T*_2_^0^,…] ∈ *R*^*N*×*s*_1_*s*_2_×*p*^. In order to learn PCA filters at the first stage, we need to obtain the eigenvectors of covariance matrix *T*^0^(*T*^0^)^*T*^ and select the largest *V*_1_ eigenvalues as the PCA filters:

(1) U1,D1=svd1N·p·V1T0T0TK1=reshapeU1,s1,s2∈Rs1×s2,

where the matrix *U*^1^ ∈ *R*^*s*_1_*s*_2_×*s*_1_*s*_2_^ contains the eigenvectors of covariance matrix and the diagonal entries of the matrix *D*^1^ holds the corresponding eigenvalues. In (1), reshape(*U*^1^, *s*_1_, *s*_2_) is a function that maps *U*^1^ ∈ *R*^*s*_1_*s*_2_^ to *U*^1^ ∈ *R*^*s*_1_×*s*_2_^. Then, with the boundary zero-padded we move on to obtain the PCA convoluted feature maps:

(2) $$\begin{array}{c} C_{ij}^{1}=\tanh\left(\;K_{j}^{1}*T_{i}^{0}\;\right)\in R^{N\times s_{1}s_{2}\times p}. \end{array}$$

Before entering into the second stage, a nonlinear transformation layer is applied using the following equation:

(3) $$\begin{array}{c} T_{ij}^{1}=a\cdot \sqrt{\varepsilon +{\left(C_{ij}^{1}\right)}^{2}}+\left(\;1-a\;\right)\cdot \log\left(\;1+e^{C_{ij}^{1}}\;\right). \end{array}$$

At the second stage, we share a similar process with stage one, and the input images of the second stage are {*T*_*ij*_^1^}_*i*=1,*j*=1_^*i*=*N*,*j*=*V*_1_^ ∈ *R*^*s*_1_*s*_2_×*V*_1_*Np*^. For *i*th image convoluted with *j*th filter and applied nonlinear transformation procedure at the previous stage, after patch mean removal we have

(4) Tij1=t¯ij,11,t¯ij,21,…,t¯ij,p1∈Rs1s2×pi=1,2,…,N,  j=1,2,…,V1.

For each input image *T*_*i*_^0^, we get *V*_1_ feature maps. So we combine them and obtain

(5) $$\begin{array}{c} {\left\{\;T_{ij}^{1}\;\right\}}_{i=1,j=1}^{i=N,j=V_{1}}=\left[\;T_{11}^{1},T_{12}^{1},\ldots ,T_{1V_{1}}^{1},T_{21}^{1},\ldots ,T_{NV_{1}}^{1}\;\right]. \end{array}$$

Similar to stage 1, we save the largest *V*_2_ eigenvalues of *T*^1^(*T*^1^)^*T*^ to get the PCA filters at the second stage, followed by convolutional layer and nonlinear transformation layer.

(6) $$\begin{array}{c} K^{2}=reshape\left(\;U^{2},s_{1},s_{2}\;\right)\in R^{s_{1}\times s_{2}}. \end{array}$$

The convoluted and nonlinear transformation layers are

(7) Cij2=tanh⁡Kj2∗Tij1∈RN×s1s2×V1NpTij2=a·ε+Cij22+1−a·log⁡1+eCij1.

#### 2.1.2. Feature Pooling

The last component of CHDNet is the feature pooling layer, containing histogram, multiscale feature analysis, high dispersal, and local response normalization. We illustrate this layer more clearly by taking a specific input image *T*_*i*_^0^ as an example.

*(a) Histogram.* For each set of feature maps, we convert feature maps belonging to the corresponding filter at the last stage into one histogram image whose every pixel is an integer in the range [0,255] and treated as a distinct “word”; *H*_*ij*_ can be expressed as

(8) Hij=∑k=1V12k−1  mod 256×HevisideTi×kj2.H¯j=Hj−min⁡Hjmax⁡Hj−min⁡Hj×255.

*(b) Multiscale Feature Analysis*. For each histogram image ${\bar{H}}_{ij}$, we constructed a sequence of grids at resolutions 0,1,…, *L*. Let ${\bar{H}}_{ij}^{l}$ denote the histogram of ${\bar{H}}_{ij}$ at resolution *l*, so that ${\bar{H}}_{ij}^{l}\left(b\right)$ is the vector containing numbers of points from ${\bar{H}}_{ij}$ that fall into the *b*th cell of the grid according to different words. We cascade ${\bar{H}}_{ij}^{l}\left(b\right)$ to build a multiscale feature map as

(9) fij=H¯ij01,H¯ij12,…,H¯ijLG∈RG×256G=∑l=0L2l,j=1,2,…,V2.

*(c) High Dispersal*. For each multiscale feature map, we use high dispersal to prevent degenerate situations and enforce competition between features by

(10) $$\begin{array}{c} {\tilde{f}}_{ij\left(x,y\right)}=\sigma \cdot \frac{f_{ij\left(x,y\right)}}{\sum_{p=1,q=1}^{r,c}{\left\|f_{ij\left(x,y\right)}\right\|}_{2}}. \end{array}$$

*(d) Local Normalization*. For each feature at the same position in different multiscale feature maps, we use local normalization to prevent redundancy by

(11) $$\begin{array}{c} {\bar{f}}_{ij\left(x,y\right)}=\frac{{\tilde{f}}_{ij\left(x,y\right)}}{{\left(\gamma +\alpha \sum_{max\left(1,j-n/2\right)}^{min\left(V_{2},j+n/2\right)}{\left({\tilde{f}}_{ij\left(x,y\right)}\right)}^{2}\right)}^{\beta }}. \end{array}$$

Finally, feature vector of the input images is then defined as

(12) $$\begin{array}{c} F_{i}={\left[\;vec\left(\;{\bar{f}}_{i1}\;\right),vec\left(\;{\bar{f}}_{i2}\;\right),\ldots ,vec\left(\;{\bar{f}}_{iV_{2}}\;\right)\;\right]}^{T}. \end{array}$$

The parameters *ε*, *a*, *γ*, *α*, *β*, *σ*, and *n* in (3), (7), (10), and (11) are determined by experimental experiences. We are using a grid search method to determine these parameters based on the randomly selected 84 training samples. To be more specific, *ε* ∈ [10^−10^, 1] with the step of 10, *a* = 0 or 1, *γ* ∈ [0,10] with the step of 2, *α* ∈ [10^−6^, 10^2^] with the step of 10, *β* ∈ [0,1] with the step of 0.25, and *σ* = (315 × *t*)/84, where *t* ∈ [0.59,0.60] with the step of 10^−2^, and *n* with the step of 1. The number of filters *V*_1_ and *V*_2_ and the size of the PCA filter *s*_1_ × *s*_2_ are decided as suggested in [23].

It should be noticed that when compared with PCANet [23], our CHDNet only shares some similarity in learning PCA kernels, but the structure of our CHDNet and especially the techniques in the feature pooling layer are new and uniquely designed to address the tongue image classification problem and significantly improve its performance.

## 3. Experiments

We used the same dataset as in our previous work [29]. Raw tongue image samples were acquired from Dongzhimen Hospital, Beijing University of Chinese Medicine. We have only collected 315 cases, and the proposed CHDNet for tongue image classification will be transformed into mobile application. More cases will be obtained by practical application. The 315 cases include 48 normal cases and 267 abnormal cases diagnosed by clinicians. Our tongue image classification consists of two steps: feature extraction and classification. Feature extraction step can be further divided into training and testing stage. During training stage of feature extraction step, in order to learn unbiased convolutional kernels, we randomly choose 40 normal and 44 abnormal samples (about 26.67% of total number of the whole tongue images in dataset) as training set, which is used to learn convolutional kernels and determine parameters *ε*, *a*, *γ*, *α*, *β*, *σ*, and *n*. With the learned kernels and determined parameters, we extract features of the left 231 samples. As a result, feature representations for 315 samples are obtained. Then these feature representations are sent into the classifier. All of the reported results in this section are the averaged outcomes after 10 rounds of 5-fold cross validation.

Besides accuracy, sensitivity, and specificity, we also use precision, recall, and *F*1-score to evaluate the performance of our proposed method and other methods. These indices are commonly used in detection literature [26]. Accuracy (ACC) = (TP + TN)/(TP + FN + FP + TN), sensitivity (SEN) = TP/(TP + FN), specificity (SPE) = TN/(TN + FP), positive predictive value (PPV) = TP/(TP + FP), negative predictive value (NPV) = TN/(TN + FN), and *F*1-score = (2 × TP)/(2 × TP + FP + FN), where

- TP (True Positive) is the number of positive samples correctly predicted by the system;
- TN (True Negative) is the number of negative samples correctly predicted by the system;
- FP (False Positive) is the number of false detection instances of positive samples by the system;
- FN (False Negative) is the number of actual positive missed by the system.

### 3.1. Impact of the Proposed Components of CHDNet

As illustrated in Section 2, our proposed CHDNet proposed modifications are based on PCANet from four aspects. The example in Table 1 shows how these new ideas improve upon the PCANet method and contribute to our final performance gain. For a fair comparison, we use LIBLINEAR SVM [30] as the classifier for all methods listed in Table 1. Here, we use the tongue images dataset and demonstrate that the combination of the proposed high dispersal (HD) method, local response normalization (LRN), multiscale feature analysis (MFA), and nonlinear transform (NT) is able to significantly improve tongue image recognition rate from 84.77% achieved by the original PCANet algorithm to 91.14%. It is well known that the misdiagnosis rate decreases with higher sensitivity, and the misdiagnosis rate decreases with higher specificity. This means that although PCANet [30] combined with LIBLINEAR SVM [30] can correctly classify the abnormal samples, it can hardly recognize the normal status. With the help of our four components, specificity improves greatly at the cost of sensitivity decreasing slightly.

### 3.2. Unbalanced Dataset Processing

In our classification task, the majority of examples are from one of the classes. The number of abnormal data is much larger than that of normal data, since most patients come to visit the hospital only when they feel ill. Unbalance in the class distribution often causes machine-learning algorithms to perform poorly on the minority class. Therefore we need to improve the performance of classifier with unbalanced dataset. In this paper, in order to achieve better performance, we adjusted the class weight of normal and abnormal samples. Since the SVM tends to be biased towards the majority class, we should place a heavier penalty on misclassified minority class.

For weighted LIBLINEAR SVM [30], we tuned the class weight for validation and training set. As we used 5-fold cross validation strategy, we partitioned the whole 315 feature representations obtained by our proposed CHDNet into 5 training and testing sets in 5 different ways. The weighted LIBLINEAR SVM [30] was first trained with 4 subsets, and then its performance was evaluated on the 5th subset. Each subset was used as the test set once through repeating the process 5 times. The final result was obtained by averaging the outcome produced in the 5 corresponding rounds. For each round, we assign weight to each class, with the majority class always set to 1 and the minority class given larger weight, namely, integers ranging from 1 to 9. Since we raised weight of the minority class, the cost of misclassifying the minority class goes up. As a result, True Positive rate becomes higher while True Negative rate turns out to be lower. To this end, in order to measure the balance of accuracy in our problem, we used the geometric mean (*g*-mean) [31] of sensitivity and specificity:

(13) $$\begin{array}{c} g=\sqrt{Sensitivity\times Specificity}. \end{array}$$

This measure has the distinctive property of being independent of the distribution of examples between classes. The result in Table 2 shows that when the weight of two classes is 8 : 1 the model outperforms other weight configurations on normal and abnormal samples.

### 3.3. Comparison of Classification Accuracy Using Different Feature Extraction Methods

During training stage, CHDNet learns convolutional kernels, and feature representations for training set can also be obtained. During testing stage, testing samples are convoluted with the PCA filters learned at training stage and applied as a nonlinear transformation. After feature pooling layer, the features that resulted from CHDNet combined with features of training set are fed into LIBLINEAR SVM [30].

According to the experimental experience [23], the number of filters is fixed to *V*_1_ = *V*_2_ = 8, and the filter size is 5 × 5. We experienced set parameters *ε* = 10^−8^, *a* = 1, *γ* = 2, *α* = 10^−4^, *β* = 0.75, *σ* = 2.238, and *n* = 5 for our CHDNet.

Table 3 shows some tongue images (with the patient numbers shown as N017, N018, D100, and X084) in our dataset and the prediction labels are based on the training LIBLINEAR SVM [30] classifier. Besides, tongue images labeled with 0 represent that patients are in normal status, while 1 reflects an abnormal gastritis condition.  Table 4 lists pathological information by TCM, which indicates that the prediction label based on our CHDNet and LIBLINEAR SVM [30] is in line with the diagnosis of the Chinese medical physician.

Sensitivity refers to the test's ability to correctly detect patients who do have the condition, and specificity relates to the test's ability to correctly detect patients without a condition. As a result, in the context of computer aided tongue image classification, we pay more attention to sensitivity and specificity than other indices (e.g., accuracy, positive predictive value, negative predictive value, and *F*1-measure) in order to find a trade-off between sensitivity and specificity.

The proposed CHDNet framework was compared with both low-level and high-level feature extracting approaches quantitatively on the same dataset under the seven different classifiers. The compared methods are single feature obtained by HOG, LBP, and SIFT, multifeatures that resulted from the combination of three mentioned single features, state-of-the-art hand-crafted features calculated by Doublets [21] and Doublets + HOG [22], and high-level features generated by PCANet. Experimental results show that the proposed feature extraction method outperformed both low-level and high-level feature extracting approaches. From Table 5, although some feature extraction methods like HOG or LBP can achieve the high sensitivity, their specificities under the same classifier which yields high sensitivity is relatively low. For example, HOG features combined with GBDT achieves 100.00% sensitivity, and the specificity is only 54.58%. If HOG features combined with a CART classifier, it can achieve 56.31% specificity, which is the best performance among the seven classifiers. However, its sensitivity is only 90.19%. That is to say, when the distribution of samples is unbalanced, the tongue images classification model based on HOG or LBP features is unbalanced. This property also holds true for other single feature extracting approaches. We can also see that multifeatures have the power of containing richer information for building a more accurate classification model when compared with single features. However, specificity is still not acceptable. While high-level feature learned through training samples can achieve the best sensitivity, they can hardly construct a balanced classification model.

**Figure 4:**
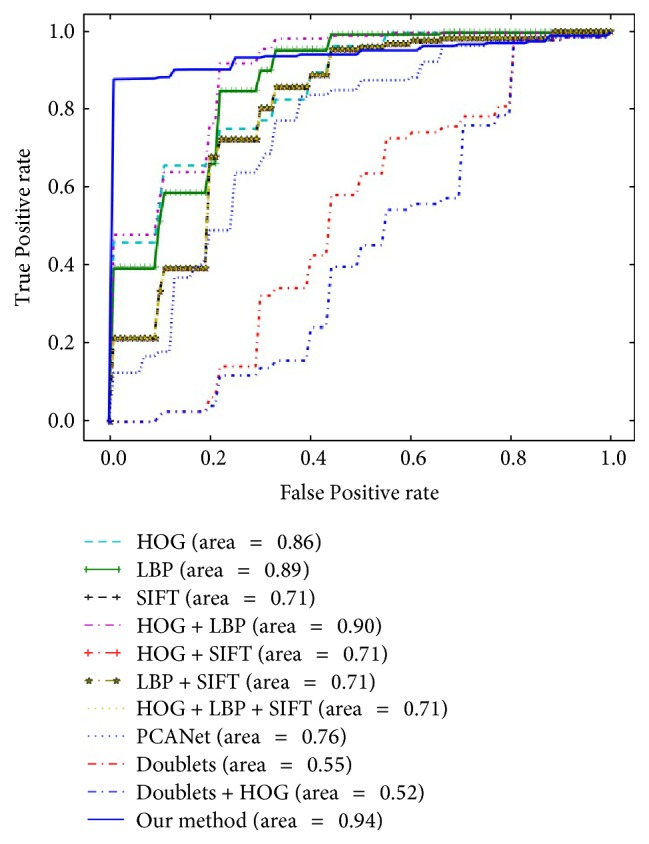
Mean receiver operating characteristic of different feature extraction methods on tongue images dataset.

As shown in Table 5, by adding nonlinear transformation, multiscale feature analysis, high dispersal, and local response normalization, our CHDNet achieved a 91.14% recognition accuracy rate, which is more than six percentage points off PCANet [23]. We noticed that the sensitivity of our proposed CHDNet is about 4.8% inferior to the best performance; however, the specificity of our CHDNet is at least 8% superior when compared to other feature extraction methods. These results indicate that the classification model based on these comparisons seems partial to the majority of the tongue image data. In addition, the receiver operating characteristic (ROC) curve is usually used to evaluate the performance of classification models. Since we repeated 5 cross validations 10 times, Figure 4 gives the mean ROC curve of each mentioned method. The bigger the area under curve (AUC), the better the performance of the model. From Figure 4, we can see the AUC of our CHDNet is equal to 0.94, which is the closest one among the compared methods. This indicates that our proposed CHDNet has the best performance when compared with the other four mentioned feature extraction methods. Even positive and negative samples are unevenly distributed.

### 3.4. Comparison of Classification Accuracy Using Different Classifiers

Another important issue for automatic classification of tongue images is to develop a high accuracy classifier. To apply deep learning models to the tongue image classification task, the problem can be considered as a feature extraction problem of digital signals or images, cascading with a classifier [2].

After obtaining feature representations, different machine-learning algorithms have been used for classification tasks. Among them, distance-based models, support vector machines (SVM), and tree based models are three widely used algorithms.

The performance of our proposed CHDNet incorporating LIBLINEAR SVM [30] was also compared with other classifiers, including LDA, KNN, CART, GBDT, RF, and LIBSVM using identical data and features. Generally, the performances of CART, RF, and GBDT are comparatively poor because the dimension of features obtained by our CHDNet is very high and these tree models are inferior to simple classifiers. Besides, since we handle classification problems well with the unbalanced distribution tongue images dataset, the performance of LDA and KNN performance is not as good as weighted LIBLINEAR SVM [30].

In bioinformatics, SVM is a commonly used tool for classification or regression purposes with high-dimensional features [32]. Instead of using LIBSVM [33] as the classifier, we use LIBLINEAR SVM [30]. The reason is that LIBLINEAR SVM [30] performs better than LIBSVM [33] when the number of samples is far smaller than the number of features. As in our CHDNet, the number of samples is 315, and the number of features of each sample is 43008. So, compared with LIBSVM [33], LIBLINEAR SVM [30] is a better choice.

As shown in Table 6, the overall performance of LIBLINEAR SVM [30] is the best of the six classifiers in terms of accuracy, specificity, precision, recall, and *F*1-score (specified in bold). After optimizing the weights of the LIBLINEAR SVM [30], the accuracy of LIBLINEAR SVM can reach 91.14%, which is 6.24% higher than LDA. Besides, the specificity of LIBLINEAR SVM [30] improves from 3% to 25% when compared with distance-based models and tree structure models. Through the comparison, we can see that SVM classifier [30, 33] with the optimal parameters is superior to the other five methods. The LIBLINEAR SVM [30] method increases the performance accuracy to 91.14% and improves other performance measurements in different levels, which are the best of all the other classifiers.

This indicates that our feature extraction framework combined with LIBLINEAR SVM [30] can be considered as a reliable indicator to normal and abnormal samples.

## 4. Conclusions

In this paper, we proposed a new framework for tongue images classification on unsupervised feature learning methods. We learned features with CHDNet and trained a weighted LIBLINEAR SVM classifier to predict normal/abnormal patients. With this novel framework, tests show that our framework combined with weighted LIBLINEAR SVM can obtain suitable features, which are able to construct the most balanced prediction model when compared with other feature extracting methods. For future study, we would like to develop a real-time computer aided tongue diagnosis system based on this approach.

## Figures and Tables

**Algorithm 1:**
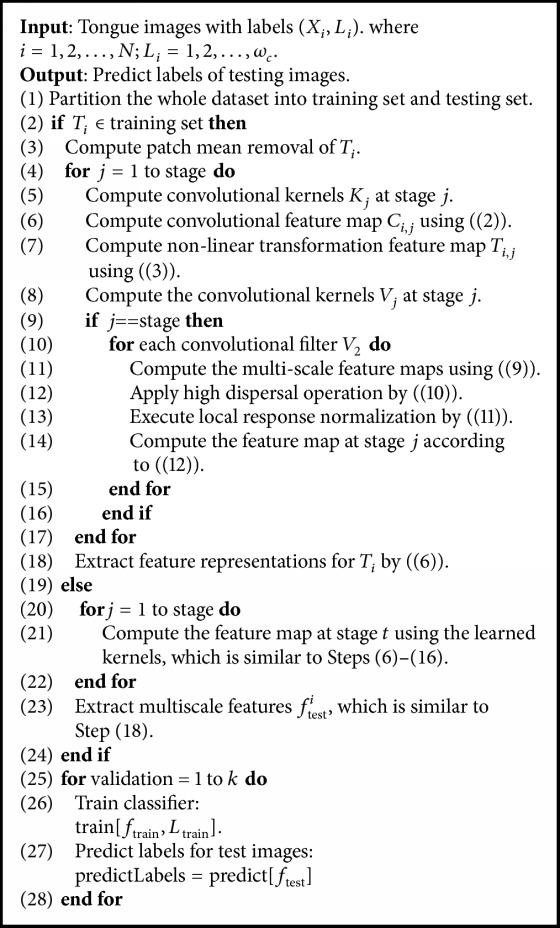
Tongue images classification based on CHDNet.

**Table 1:** Performance comparison on the tongue images dataset with the proposed components of CHDNet.

Method	Accuracy	Sensitivity	Specificity
PCANet	84.77%	100.00%	0.00%
PCANet + NT	85.40%	100.00%	4.17%
PCANet + MFA	86.01%	99.25%	12.31%
PCANet + HD	87.37%	98.16%	27.35%
PCANet + LRN	84.77%	100.00%	0.00%
CHDNet with all four components	91.14%	94.26%	75.40%

**Table 2:** Performance comparison for weighted LIBLINEAR SVM.

Normal : Abnormal	Accuracy	Sensitivity	Specificity	*G*-mean
*w*1 : *w*0 = 1 : 1	90.61%	93.79%	72.93%	68.40%
*w*1 : *w*0 = 2 : 1	91.01%	94.22%	73.36%	69.12%
*w*1 : *w*0 = 3 : 1	90.62%	94.57%	68.60%	64.88%
*w*1 : *w*0 = 4 : 1	90.94%	94.19%	72.89%	68.66%
*w*1 : *w*0 = 5 : 1	91.02%	94.79%	70.07%	66.42%
*w*1 : *w*0 = 6 : 1	90.55%	94.28%	69.89%	66.25%
*w*1 : *w*0 = 7 : 1	90.59%	94.87%	66.91%	63.48%
*w*1 : *w*0 = 8 : 1	91.14%	94.26%	75.40%	71.07%
*w*1 : *w*0 = 9 : 1	91.04%	94.39%	72.27%	68.22%

**Table 3:** Some normal and abnormal tongue images classified by our method.

Patient's number	Original image	Mask	Tongue body	Normalization	Predict label	Actual label
N017	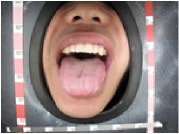	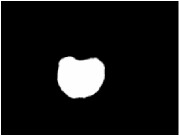	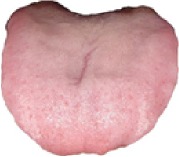	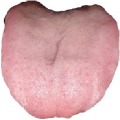	0	0
N018	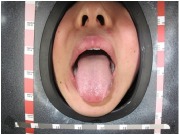	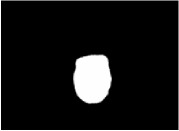	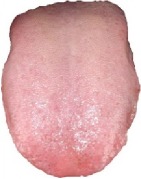	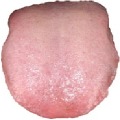	0	0
D100	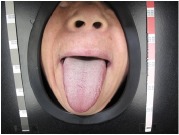	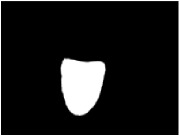	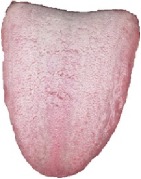	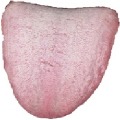	1	1
X084	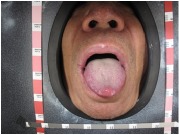	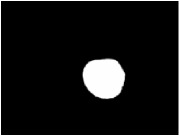	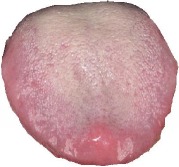	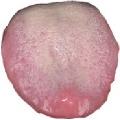	1	1

The first column is the original image of 1728 × 1296 pixels; the second column is the background mask; the third column is the extraction of tongue body; the fourth column is the color space transformed tongue body image; the fifth column is the normalized 512 × 512 pixels image; the last column is the predicted label.

**Table 4:** Pathological information by TCM.

Patient's number	Pathological feature	Clinical practitioner's subjective diagnosis	Diagnosis result
N017	—	—	Normal

N018	—	—	Normal

D100	Superficial gastritis	Cold Zheng-deficiency cold of the spleen	Abnormal

X084	Atrophic gastritis	Hot Zheng-damp heat in the spleen and the stomach	Abnormal

The first column is the patient's number, the second column is pathological feature, the third column is clinical practitioners subjective diagnosis, and the last column is the clinical practitioners diagnosis result.

**Table 5:** Comparison of the proposed method with other feature extracting approaches.

		LDA	KNN	CART	GBDT	RF	LIBSVM	LIBLEAR SVM
HOG [20]	Sensitivity	91.65%	100.00%	90.19%	100.00%	100.00%	99.93%	98.02%
	Specificity	58.00%	22.33%	56.31%	54.58%	47.42%	48.82%	51.27%
LBP	Sensitivity	99.70%	100.00%	90.49%	92.06%	100.00%	100.00%	100.00%
	Specificity	64.56%	60.93%	65.91%	65.16%	58.29%	58.64%	58.84%
SIFT	Sensitivity	98.95%	100.00%	90.49%	92.78%	100.00%	98.28%	98.17%
	Specificity	41.44%	0.20%	58.18%	55.20%	62.31%	44.53%	45.42%
HOG + LBP	Sensitivity	99.93%	99.96%	91.38%	92.85%	100.00%	100.00%	100.00%
	Specificity	49.60%	56.69%	64.49%	67.24%	58.20%	60.33%	60.36%
HOG + SIFT	Sensitivity	100.00%	100.00%	91.87%	92.32%	100.00%	98.19%	97.76%
	Specificity	59.91%	0.42%	60.56%	58.29%	62.49%	43.87%	46.62%
LBP + SIFT	Sensitivity	100.00%	100.00%	91.58%	91.90%	100.00%	98.31%	97.87%
	Specificity	58.89%	0.82	62.31%	63.33%	60.20%	44.07%	44.33%
HOG + LBP + SIFT	Sensitivity	99.96%	100.00%	91.95%	92.85%	100.00%	98.27%	98.24%
	Specificity	59.67%	0.87%	65.64%	66.33%	59.93%	43.18%	46.18%
Doublets [21]	Sensitivity	91.88%	100.00%	93.22%	93.67%	100.00%	100.00%	100.00%
	Specificity	36.71%	0.00%	48.91%	49.42	25.51%	0.00%	0.00%
Doublets + HOG [22]	Sensitivity	92.35%	100.00%	94.22%	94.38%	100.00%	100.00%	100.00%
	Specificity	29.96%	0.00%	51.80%	46.44%	27.29%	0.00%	0.00%
PCANet [23]	Sensitivity	100.00%	98.35%	87.05%	88.50%	100.00%	100.00%	100.00%
	Specificity	0.00%	14.09%	29.51%	28.40	0.00%	0.00%	0.00%
Our method	Sensitivity	90.68%	93.11%	92.44%	92.63%	93.37%	94.68%	94.26%
	Specificity	52.91%	69.18%	61.56%	59.98%	65.62%	71.18%	75.40%

*Note*. The sum rule of feature combination is a cascade operation. Given two types of features *f*_*i*_ and *f*_*j*_ obtained by feature extraction methods FEM_*i*_ and FEM_*j*_, respectively, then FEM_*i*_ + FEM_*j*_ is equal to [*f*_*i*_, *f*_*j*_]. For example, HOG + LBP means, for each sample, we append LBP features just after HOG features. Besides, “our method” is short for the proposed CHDNet feature extraction method.

**Table 6:** Comparison of the proposed method with different classifiers.

Classifier	ACC	SEN	SPE	PPV	NPV	*F*1-score
LDA	84.90%	91.09%	50.42%	91.16%	51.10%	91.08%
KNN	89.55%	93.10%	69.71%	94.56%	65.18%	93.78%
CART	87.67%	93.03%	57.80%	92.56%	60.04%	92.75%
GBDT	87.91%	93.18%	58.60%	92.65%	62.72%	92.87%
RF	88.92%	93.29%	64.60%	93.70%	64.65%	93.45%
LIBSVM	91.27%	94.76%	72.04%	95.00%	72.90%	94.83%
LIBLINEAR SVM	91.14%	94.22%	75.40%	95.59%	74.56%	94.83%

*Note*. ACC = accuracy, SEN = sensitivity, SPC = specificity, PPV = positive predictive value, and NPV = negative predictive value.
